# On the Need for New Measures of Phylogenomic Support

**DOI:** 10.1093/sysbio/syac002

**Published:** 2022-02-17

**Authors:** Robert C Thomson, Jeremy M Brown

**Affiliations:** 1 School of Life Sciences, University of Hawai‘i, 2538 McCarthy Mall, Edmondson Hall 216, Honolulu, HI 96822, USA; 2 Department of Biological Sciences and Museum of Natural Science, Louisiana State University, Baton Rouge, LA, USA

## Abstract

The scale of data sets used to infer phylogenies has grown dramatically in the last decades, providing researchers with an enormous amount of information with which to draw inferences about evolutionary history. However, standard approaches to assessing confidence in those inferences (e.g., nonparametric bootstrap proportions [BP] and Bayesian posterior probabilities [PPs]) are still deeply influenced by statistical procedures and frameworks that were developed when information was much more limited. These approaches largely quantify uncertainty caused by limited amounts of data, which is often vanishingly small with modern, genome-scale sequence data sets. As a consequence, today’s phylogenomic studies routinely report near-complete confidence in their inferences, even when different studies reach strongly conflicting conclusions and the sites and loci in a single data set contain much more heterogeneity than our methods assume or can accommodate. Therefore, we argue that BPs and marginal PPs of bipartitions have outlived their utility as the *primary* means of measuring phylogenetic support for modern phylogenomic data sets with large numbers of sites relative to the number of taxa. Continuing to rely on these measures will hinder progress towards understanding remaining sources of uncertainty in the most challenging portions of the Tree of Life. Instead, we encourage researchers to examine the ideas and methods presented in this special issue of *Systematic Biology* and to explore the area further in their own work. The papers in this special issue outline strategies for assessing confidence and uncertainty in phylogenomic data sets that move beyond stochastic error due to limited data and offer promise for more productive dialogue about the challenges that we face in reaching our shared goal of understanding the history of life on Earth.[Big data; gene tree variation; genomic era; statistical bias.]

Phylogenetic inference is fundamentally a problem of statistical estimation. By gathering data, in this case recording the characteristics of organisms that are the products of evolutionary change and shared inheritance along an unobserved tree, we can draw inferences about the nature of the tree itself. We can estimate who the common ancestors of these organisms were, how much change occurred in their traits, and how long different lineages persisted. However, statistical estimation is challenging, and the translation of character data to knowledge about the underlying tree can be error prone. Therefore, as with any other type of statistical procedure, we need not only an estimate of a tree but also a measure (or measures) of our confidence in it. But how do we best do this?

Almost every answer to the question of how confident we should be in a phylogenetic tree has relied on one of two measures of statistical support: the nonparametric bootstrap proportion (BP; Felsenstein 1985) or the posterior probability (PP; Rannala and Yang 1996; Yang and Rannala 1997; Larget and Simon 1999; Mau et al. 1999) of the individual bipartitions that comprise the phylogeny. If these measures are close to one for all or most bipartitions in the tree, then it is common practice to describe the evolutionary history under study as “well resolved.” While arising from different statistical frameworks and involving different algorithms for their calculation, both BPs and PPs capture uncertainty in a similar way. They tell us how confident we should be in our tree if an error in our estimate is primarily due to having a limited amount of data. They both also assume that any potential source of variation in phylogenetic signal across sites or genes is well understood and captured in the assumptions of our models.

While these measures have served as important indicators of phylogenetic progress for many years, we argue that they have outlived their utility as the *primary* means of measuring phylogenetic support for modern, phylogenomic data sets with large numbers of sites relative to the number of taxa. The reason for this is that, over the last two decades, the scale at which our field is able to amass data has increased by several orders of magnitude owing to advances in both molecular sequencing and phenotyping technologies. This technological development has gradually transformed these two common measures of phylogenetic support from being reasonable ways to describe uncertainty in a phylogenetic analysis to nearly meaningless statistics that primarily reflect data set size. Continued primary reliance on these measures hinders communication, decreases the scientific merit of our field, and obscures rather than clarifies what are now the most important sources of uncertainty in modern phylogenomic analyses.

## The Issue

BPs and marginal PPs of bipartitions are both estimated as frequencies with which a bipartition appears in a set of trees. In the former case, we take the frequency that the bipartition appears in the set of maximum-likelihood trees estimated from each of many pseudoreplicated data sets (Felsenstein 1985). In the latter, we take the bipartition’s frequency in the set of trees sampled from the Bayesian marginal PP distribution of phylogenetic topologies (Larget and Simon 1999). Accordingly, both measures depend critically on the amount of variation that we observe within these particular tree sets. Highly variable tree sets will lead to measures of support that are small, while invariable tree sets mean that these measures will take on their maximal value of 1 (for all bipartitions found in the tree) or 0 (for all possible bipartitions not found in the tree).

These procedures have been successfully and widely applied as our field has developed, and they are clearly useful to the extent that what they are intended to measure, stochastic error due to limited amounts of data, is an actual issue for the data set under study (Hillis and Bull 1993; Huelsenbeck and Rannala 2004). However, as data sets grow large, stochastic error declines, and so these measures are expected to take on their maximal (or minimal) values for many “typical” phylogenomic data sets that we study today. Critically, miniscule stochastic error and correspondingly large measures of support do little to inform us about unexpected heterogeneity in a data set, to indicate when problems may exist with data quality, to flag correlations between phylogenetic signal and properties of different loci, nor to highlight potential violations of model assumptions. Instead, large support values may even be exacerbated by such problems in the analysis.

By sampling hundreds or thousands of markers across the genome, phylogenomic data sets capture DNA sequences that have different histories and have evolved under varying molecular processes. Nonetheless, phylogenomic analyses typically make strong assumptions about the nature of both gene tree heterogeneity and molecular evolutionary processes. Gene tree variation is often assumed to have been driven by a single process (e.g., the multispecies coalescent) or absent altogether. Ongoing development of more complex models (e.g., the multispecies network coalescent) is beginning to alleviate this limitation, although restrictive assumptions are still generally required. Standard models of molecular evolution also usually assume that all, or at least large subsets, of the data have evolved with the same evolutionary dynamics (i.e., that the data are independent and identically distributed). There is often little reason to suspect that this assumption holds, which has motivated the development of several approaches that allow it to be partially relaxed (e.g., Yang 1994). Beyond variation among loci or sites, exceedingly few empirical phylogenomic analyses even attempt to consider heterogeneity in evolutionary processes across branches of the tree. These biological sources of heterogeneity aside, the assembly of phylogenomic data sets also usually relies on highly automated procedures (e.g., the assignment of orthologs and paralogs, alignments) and even small error rates in some of these can change phylogenetic conclusions with maximal statistical support (e.g., Brown and Thomson 2017).

Approaches for realistically modeling the heterogeneity that the data contain unfortunately do not scale easily to genomic data sets. As data sets grow large, heterogeneity in the evolutionary process among sites and loci must also grow. Our best inference models are generally not flexible enough to capture this, which means that the models we use to fit the data may become more and more oversimplified relative to the data as more of the genome is sampled. Oversimplified models increase the risk of bias, which means that the analysis will suggest that more confidence (higher statistical support) is warranted in the result than is appropriate (Kumar et al. 2012).

Because the stochastic error is typically small (leading to high measures of support) and concern about statistical bias arising from overly simplistic models is substantial (also leading to high measures of support), the observation that virtually all phylogenomic analyses recover maximal support values is unsurprising and tells us little that we did not already know. For this reason, the time has come to broaden how we measure statistical support for modern analyses.

## The Need for New Approaches

Continued reliance on these measures in cases where we already know what they will say causes several problems. Most concerning among these is that they lead us away from clarity in interpreting results. A newly reported phylogenetic result that recovers maximal support values on all nodes may lead readers to the view that the tree is well understood, the data are powerful, and nothing is amiss. However, because maximal support values are so commonly expected for large data sets, readers are left with no way to distinguish between those trees that are strongly supported and well-established from those that remain contentious. A far better way to approach the interpretation of phylogenomic results is to begin with the expectation that these measures will take on their maximal values and plan to assess other important sources of uncertainty in the analysis, in addition to the narrow sense of uncertainty that BPs and PPs capture. This approach would lead to a richer, more thoughtful, and more useful discussion of uncertainty in phylogenomic analyses.

Continued reliance on these measures also does little to advance the primary goal of our field. That central goal, inference of a highly complete and well-understood evolutionary history of life on Earth, rests on the central idea that there is one single evolutionary history of life (even if it might be bewilderingly complex and difficult to infer). As biological sampling, data collection, and statistical methods all improve, we would hope to see consilience emerge among phylogenetic results as they become more accurate depictions of this singular evolutionary history. Indeed, we have seen this for much of the tree of life, and it makes sense to continue focusing on it as trees become more comprehensive. However, current practice does little to help us do this. For instance, consider how phylogenetic studies often place their results in the context of earlier work. In analyzing a new large data set, we may find that clade A is monophyletic with a BP of 1.0. To put this in context, we might summarize previous work by pointing out that earlier papers looking at the members of clade A did not find monophyly, or did so with a smaller BP. Typically, those earlier papers will employ a smaller data set than our current study, which means that their BP (as a measure of sample variance) is likely to be smaller than ours. The higher BP in our current study would say something about the progress that has been made by collecting more data. However, if we expect the BP to be 1.0 for the large data set, irrespective of what particular monophyletic groups we recover, comparisons like these are not particularly meaningful.

Similarly, some nodes in the tree of life that have proven difficult to resolve have accumulated several repeated studies all employing large data sets that are carefully analyzed. This, at least occasionally, leads to a set of papers that say fundamentally different things about the node under study, but that are maximally supported by traditional support measures (e.g., Brown and Thomson 2017; Reddy et al. 2017; Li et al. 2021). Clearly, in such cases, these measures are not providing a true assessment of uncertainty regarding these nodes. Taking an example from our own work, a PP of 1.0 that turtles are sister to the alligators and crocodiles does not come close to the meaning we are 100% confident that this is the true relationship, because the many other papers that examine this clade find, often with similarly maximal support, that turtles are sister to lepidosaurs, or sister to archosaurs, or sister to literally every other major clade of amniotes except mammals (Brown and Thomson 2017). The most important source of uncertainty regarding the phylogenetic position of turtles has to do with an explanation of these strongly supported but incompatible results, not the stochastic error associated with any one study. If we care to resolve the phylogenetic position of turtles accurately, we need to focus on why different studies arrive at different conclusions. Why do repeated large samples from the genome so frequently lead us to conflicting conclusions in phylogenetics? Progress in our field is directly linked to our ability to answer this question, but it is one that is rarely addressed in modern analyses.

## New Approaches

A growing group of researchers have recognized the need to extend methods for measuring and reporting on phylogenetic support for large data sets, and we are happy to introduce a set of creative approaches for doing so here. This collection of papers, and several more that precede them in the literature (e.g., Goldman 1993; Bollback 2002; Brown 2014; Allman et al. 2017; Arcila et al. 2017; Brown and Thomson 2017; Walker et al. 2018; Minh et al. 2020; see also several more discussed by Simon 2022 in this issue), represent important steps toward a modernized way of thinking about and quantifying statistical support in phylogenomic analysis. They are sure to provide a useful set of ideas for empiricists working today, and we hope that they will inspire more work in this area.

The special issue begins with a Historical Essay by Simon (2022) that provides a comprehensive overview of current approaches for measuring bipartition support, including their development and ongoing work to extend their utility. Arcila et al. (2021) apply many of these support metrics in order to understand how they compare to one another and what lessons this may provide for phylogenomics going forward. The papers from Shen et al. (2021) and Walker et al. (2022) interrogate the extensive heterogeneity and conflict present in phylogenomic data, exploring both how such conflict manifests in measures of support and how to accommodate it. Allman et al. (2022) are similarly interested in heterogeneity across loci and develop a graphical method for summarizing it, showing how it can be used to measure whether heterogeneity is well-described by available models—the multispecies coalescent, in this case. Naser-Khdour et al. (2022) make use of long-available, but little explored, nonreversible models of substitution and demonstrate their utility for estimating the root placement of phylogenetic trees with support using genome-scale data. Mount and Brown (2022) explore the utility of likelihood ratios for understanding genome-wide patterns of concordance and conflict, undertaking a comparison of maximal versus marginal likelihoods to illustrate the tradeoffs among the perspectives these measures provide. While this special issue focuses largely on issues that arise as a result of the heterogeneity contained in data sets with many sites, the phylogenomic era has also made it possible to completely sequence large numbers of small genomes (e.g., viral genomes in a pandemic) and such data sets come with their own statistical challenges. The paper by Wertheim et al. (2022) explores some of these challenges in the case of viral phylogenomic data sets where, despite sampling whole genomes, the number of variable sites remains small and data sets may be close to homoplasy free. Our hope is that this special issue will encourage researchers to embrace the many and varied statistical challenges that come along with genome-scale data, employing careful means to measure and describe them, and helping the field to embrace the challenge of statistical estimation in the phylogenomic era.
